# Reactivation cytomegalovirus leading to acute myocardial infarction—A first reported case in an immunocompetent patient

**DOI:** 10.1002/ccr3.3914

**Published:** 2021-02-10

**Authors:** Zohaib Yousaf, Nadeen Albaz, Alaaedin A. Abdelmajid, Taher Sabobeh, Abdel‐Naser Elzouki

**Affiliations:** ^1^ Department of Medicine Hamad Medical Corporation Doha Qatar; ^2^ Infectious Diseases Hamad General Hospital Hamad Medical Corporation Doha Qatar; ^3^ College of Medicine Qatar University Doha Qatar; ^4^ Weill Cornell Medical College Doha Qatar

**Keywords:** CMV, colitis, hepatitis, myocardial infarction, reactivation

## Abstract

Reactivation of cytomegalovirus (CMV) in immunocompetent patients may lead to increase morbidity and mortality. A clinical suspicion allows timely diagnosis, treatment, and favorable outcome. In a subject without apparent risk factors for acute myocardial infarction (AMI), CMV can be a possibility.

## Graphical abstract

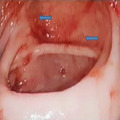

## 1 INTRODUCTION

Cytomegalovirus (CMV) is a DNA virus belonging to herpesvirus. It modulates host immunity and may cause immune dysfunction leading to immunosuppression and auto‐immune phenomenon.^1^ CMV can affect the functioning of both innate and adaptive immune responses by the destruction of crucial immune substrate, alteration of transcriptional and translational control, alteration of signal transduction cascades, and the production of novel interfering proteins.^2^ CMV can infect endothelial cells in vitro with certain strains leading to complete lysis of the endothelial cells.^3^ Infection with CMV can modulate the activity of the endothelium‐from anticoagulant to procoagulant state.^4^, ^5^ It can also induce platelet adherence and aggregation in infected endothelium, with acute‐phase infections associated with thrombosis.^6^ CMV infection is associated with the development of atherosclerosis in the coronary arteries.^7^ We discuss a unique case of a young immunocompetent female patient with no apparent cardiovascular risk, presenting with acute myocardial infarction, with evidence of acute CMV infection.

## 2 CASE PRESENTATION

A 38‐year‐old, previously healthy female patient experienced dizziness followed by loss of consciousness for a few seconds. Two days before the loss of consciousness, she experienced a gradual onset of intermittent left‐sided chest pain, shortness of breath, palpitations, upper abdominal pain, and vomiting. During the initial evaluation, she became unresponsive and pulseless. Cardiopulmonary resuscitation (CPR) was initiated, and the patient was found to be in ventricular fibrillation. There was a return of spontaneous circulation after a single DC‐shock and 6 minutes of CPR.

**FIGURE 1:**
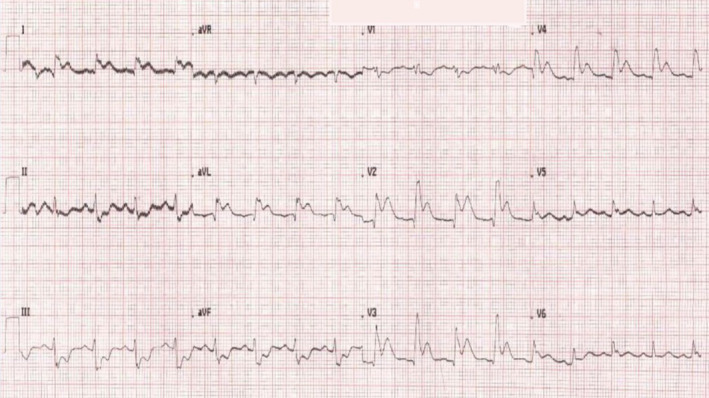
Electrocardiogram showing ST‐elevation in the anterolateral leads

Following CPR, her electrocardiography showed ST‐elevation in the anterolateral leads (Figure 1). Primary percutaneous coronary intervention protocol was activated; however, she developed ventricular fibrillation before she could be transferred for primary percutaneous coronary intervention. She had six cardiac arrests with ventricular fibrillation, each one lasting successively longer than the prior episode with return of spontaneous circulation between the episodes. A decision was made in conjunction with the cardiology and critical care team for thrombolytic therapy during CPR using r‐tPA (recombinant‐tissue plasminogen activator). She received a total of 14 DC shocks, 900 mg of amiodarone, and adrenaline and nor‐adrenaline infusions. After 180 minutes of the initial arrest, veno‐arterial extracorporeal cardiopulmonary resuscitation (VA e‐CPR) was initiated, and clinical hypothermia was induced, leading to hemodynamic stability. The patient received aspirin, clopidogrel, atorvastatin, and heparin infusion. Repeated electrocardiography showed a complete resolution of ST‐segment elevations.

Initial blood tests at time of admission were unrevealing except for a raised troponin of 189 ng/L (Table 1). On day 2 of her hospital stay, she developed nonfluid responsive hypotension requiring two concurrent vasopressors and was found to have a hemoglobin of 5.5 gm/dL. Tri‐phasic CT‐chest, abdomen, and pelvis were suggestive of hemoperitoneum and colon ischemia. Urgent exploratory laparotomy showed multiple small omental bleeds with lacerations. Hemostasis was achieved, and two drains were applied. The patient had four relook surgeries and was found to have dark discoloration of hepatic flexure of the colon without bowel perforation. She also developed acute kidney injury secondary to acute tubular necrosis requiring five sustained low‐efficiency dialysis sessions. The patient had extracorporeal membrane oxygenation (ECMO) de‐cannulation on day 6.

**TABLE 1:** Laboratory investigations at time of admission and during receiving the course of antiviral treatment

	Admission (Day 0)	During antiviral therapy^a^ (Day 35)	During follow‐up (Day 70)	Normal range
WBC	12.1	12.4	5.3	4‐11 × 10^9^/L
Hb	11.7	9.8	9.2	12‐15 g/dL
Platelet	100	533	250	140‐450 × 10^5^/L
INR	1.1	1.2	1.2	‐
BUN	3	7.4	5.9	2.1‐8 mmol/L
Creatinine	117	74	62	44‐100 mmol/L
Na	139	133	139	135‐145 mmol/L
K	3.5	5.5	4.1	3.5‐5.2 mmol/L
Cl	105	98	109	96‐106 mmol/L
HCO3	12.3	19	19.6	22‐29 mg/dL
ALP	67	730	128	35‐104 U/L
ALT	326	372	42	5 ‐ 41 U/L
AST	351	224	23	5‐ 40 U/L
Total Bilirubin	4	16	3.7	<5.1 mol/L
CRP	2.2	116	9	<5 mg/L

^a^ Intravenous ganciclovir 5 mg/kg twice daily for two weeks followed by oral valganciclovir 900 mg twice daily for 8 weeks.

**FIGURE 2:**
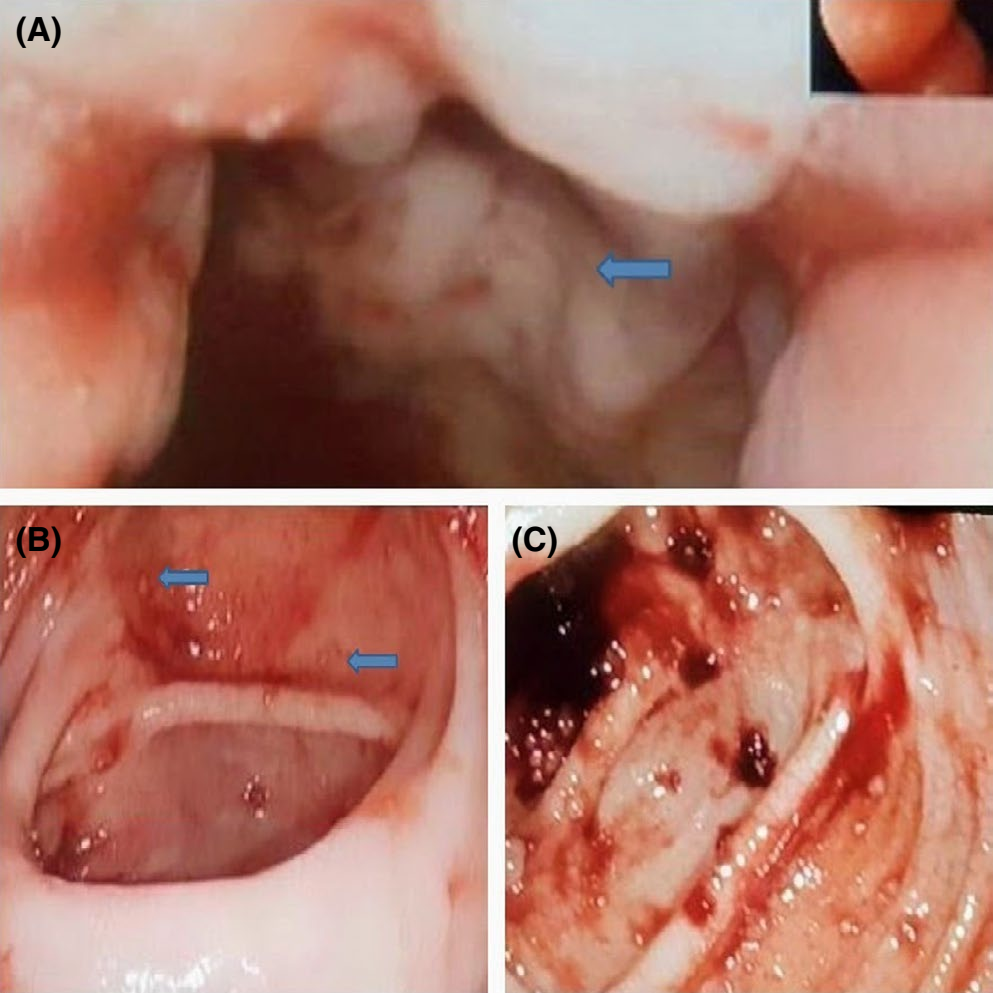
A, B, and C, Colonoscopy showing multiple large circumferential ulcers seen in ascending colon extending to the proximal transverse colon

**FIGURE 3:**
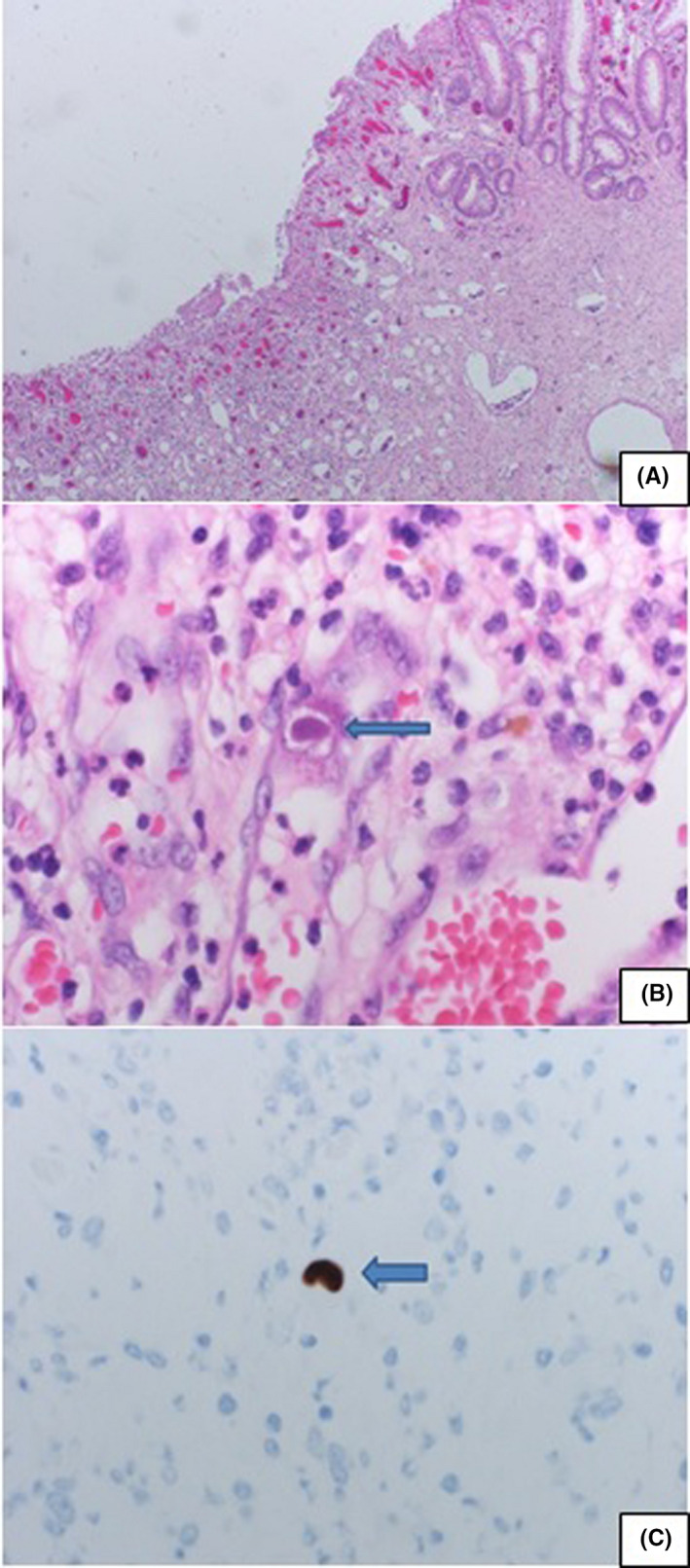
A, Low power view of colonic mucosa showing extensive mucosal ulceration with granulation tissue formation on the left. Some preserved mucosal crypts on the right upper corner. B, High power view with CMV inclusion within an endothelial cell, lining the mucosal capillary within the ulcer base's granulation tissue. Basophilic intranuclear inclusion bodies (Cowdry bodies) surrounded by a clear halo. C, High power view showing immunohistochemical stain for CMV shows positive staining, confirming the diagnosis

She developed massive gastrointestinal bleeding with a hemoglobin drop from 10.2 gm/dL to 4.1 gm/dL. A massive blood transfusion protocol was activated. Colonoscopy showed multiple large circumferential ulcers that were injected with epinephrine (Figure 2A‐C). Clipping attempt at the underlying vessel failed, and a right hemicolectomy with end ileostomy was performed. The biopsy of the colonic ulcers showed CMV colitis (Figure 3A‐C). Intravenous ganciclovir 5mg/Kg twice daily was initiated with a positive CMV polymerase chain reaction (PCR). Two weeks later, repeated CMV‐PCR was negative. The patient was switched to oral valganciclovir 900mg twice daily for 8weeks. Table 1 shows laboratory investigations during receiving antiviral therapy.

Coronary angiography revealed left anterior descending (LAD) segmental single‐vessel disease with plaque. There was a successful insertion of a single drug‐eluting stent in the LAD.

The patient was discharged and received twice‐weekly physiotherapy sessions for 8 weeks postdischarge. A reversal of ileostomy was done at five months from its creation. Clopidogrel was discontinued at one‐year postangiography, and repeat echocardiography showed an ejection fraction of 56%. The patient retained an excellent functional status with no neurological deficit, anginal symptoms, palpitations, or dizziness. She continues to follow‐up with cardiology and general surgery on a 6‐monthly basis.

### 2.1 Methods and procedures

The patient had a complicated hospital course. Determining the etiology for cardiac arrest was a challenge for the multidisciplinary team. A CT‐pulmonary angiogram ruled out pulmonary embolism. A transthoracic echocardiogram showed no intracardiac thrombus or valve vegetation and a 48% ejection fraction. A 72‐hour holter was normal.

A family history of cardiomyopathy, CAD, sudden cardiac death, or any inborn errors of metabolism was negative. There was no history of smoking, alcohol intake, illicit drug use, over‐the‐counter pills, supplements, or herbal medications.

Traditional risk factors of CAD, including dyslipidemia, diabetes, hypertension, or hypercoagulability, were absent. The hypercoagulability workup revealed no abnormality in PT, aPTT, fibrinogen, protein C, protein S, Antithrombin III deficiency, factor V Leiden, and homocysteine levels.

An extensive workup for any cause of immunocompromise was unrevealing except for the ANA panel. HIV antibodies and serology for hepatitis A, B, and C were all negative. ANA titer was 1:160, and repeated titer was 1:40. The patient had no other clinical or laboratory features suggestive of auto‐immune diseases. Anticardiolipin, Lupus anticoagulant, ANCA, antidouble stranded‐DNA, anti‐Jo, anti‐Ro, anti‐LA, anti‐RNP, anti‐Scl‐70, anti‐Smith, C3, and C4 were unremarkable.

The CMV IgG and IgM were elevated along with high serum CMV‐PCR titers. The histopathological examination of the colon showed active CMV colitis. Also, of note are the patient's liver enzymes found elevated on a regular health screen before the cardiac arrest. The liver enzymes started to improve with ganciclovir treatment. Coronary angiography showed a complete LAD plaque with small thrombus leading to occlusion.

## 3 DISCUSSION

We hypothesize a chronic CMV infection leading to endothelial dysfunction and a procoagulant state followed by reactivation leading to acute occlusion and cardiotoxicity. It is postulated that CMV infection induces the progression of atherosclerosis in CAD. A meta‐analysis of 55 studies showed individuals with positive CMV IgM antibody by enzyme‐linked immunosorbent assay or positive PCR were 1.67 times more likely to develop CAD, especially in Asian population.^8^ In a study of 105 patients who underwent coronary artery bypass grafting surgical interventions, Izadi et al reported that about 26% of these patients had positive testing of CMV‐PCR in the coronary artery plaques, the patients who had positive family history of CAD or has suffered from acute coronary syndrome (ACS) before were more likely to have positive CMV‐PCR with statistical significance.^9^ The mechanism of this relation is still not fully understood, but it was proposed that CMV can alter the cellular signal transduction of second messengers to alter cellular functions.^10^ It also enhances the proliferation of smooth muscle cell which might contribute to the etiology of CAD. Furthermore, CMV infection can temporarily increase the level of antiphospholipid antibodies, thus increasing the risk of acute thrombosis.^11^

CMV can affect immune‐competent individuals with a variety of severe clinical syndromes. While many case reports of CMV hepatitis, myelitis, colitis, and myo‐pericarditis were described,^12^ but to the best of our knowledge, no reported case describing a young immune‐competent individual presenting with ACS possibly attributed to CMV infection, and without known risk factors of CAD, has been described previously.

Our patient's coronary angiography showed a complete LAD coronary artery occlusion which leads to the cascade of events. Although it is reported that acute CMV infection is associated with acute thrombosis, most common thrombosis sites, according to a meta‐analysis, were deep vein thrombosis/pulmonary embolism in immune‐compromised patients and splanchnic vein thrombosis and splenic infarction in immune‐competent individuals.^13^ Considering the elevated IgG and IgM CMV‐antibodies in our patient, we propose a chronic CMV infection leading to atherosclerosis followed by reactivation leading to acute occlusion and myocardial infarction.

It is noteworthy that the histopathological examination of our patient’ resected colon segment showed active CMV colitis. Also of mark that the patient's liver enzymes were elevated on initial presentation even before the cardiac arrest and did not improve till ganciclovir treatment started which is against ischemic hepatitis and suggests probable CMV hepatitis.

A wide panel of investigations to reveal whether the patient was immunocompromised were conducted, all of which reported back unremarkable except for the positive ANA panel which its titer improved after antiviral therapy. The patient had no other clinical or laboratory features suggestive of systemic lupus erythematosus or any other auto‐immune diseases. Possible explanations of this finding include CMV direct damage to endothelial cells and molecular mimicry, inducing autoantibodies’ production.^14^, ^15^

## 4 CONCLUSION

To best of our knowledge, this report is the first describing a possible association between CMV and acute myocardial infarction in a young immunocompetent patient, who also had CMV colitis and CMV hepatitis. Reactivation of CMV infection in immunocompetent patient may lead to increase morbidity and mortality by affecting multiple organ systems including heart, colon, and liver. A high clinical suspicion allows immediate diagnosis and treatment and eventually a favorable outcome. This case report may suggest the need for further studies to assess whether early detection and treatment of CMV infection will lead to improve outcomes.

## WRITTEN INFORMED CONSENT

Written informed consent was obtained from the patient for publication of this case report and accompanying images. The patient understand that her name and initials will not be published and all due efforts will be made to conceal her identity.

## CONFLICTS OF INTEREST

The authors have no conflict of interest to declare.

## AUTHORS’ CONTRIBUTIONS

ZY: involved in manuscript writing, literature review, manuscript revisions, and approval of the final manuscript. NA: involved in manuscript writing, literature review, and approval of the final manuscript. AA: involved in manuscript writing, literature review, and approval of the final manuscript. TS: involved in literature review and approval of the final manuscript. AE: involved in case identification, manuscript writing, critical review, and approval of the final manuscript.

## ETHICAL APPROVAL

Ethical approval for the manuscript has been obtained from the medical research council of Hamad Medical Corporation with the manuscript number MRC‐04‐20‐14.

## Data Availability

Data sharing not applicable to this article as no datasets were generated or analyzed during the current study.
